# An Insight into the Role of Postmortem Immunohistochemistry in the Comprehension of the Inflammatory Pathophysiology of COVID-19 Disease and Vaccine-Related Thrombotic Adverse Events: A Narrative Review

**DOI:** 10.3390/ijms222112024

**Published:** 2021-11-06

**Authors:** Chiara Stassi, Cristina Mondello, Gennaro Baldino, Luigi Cardia, Alessio Asmundo, Elvira Ventura Spagnolo

**Affiliations:** 1 Legal Medicine Section, Department for Health Promotion and Mother-Child Care, University of Palermo, Via del Vespro, 129, 90127 Palermo, Italy; chiara_stassi@libero.it (C.S.); gennarobld@hotmail.it (G.B.); 2 Department of Biomedical and Dental Sciences and Morphofunctional Imaging, University of Messina, Via Consolare Valeria, 1, 98125 Messina, Italy; aasmundo@unime.it; 3 Department of Clinical and Experimental Medicine, University of Messina, Via Consolare Valeria, 1, 98125 Messina, Italy; luigicardia1@gmail.com

**Keywords:** COVID-19, SARS-CoV-2, pathophysiology, vaccine, ChAdOx1 nCov-19, immunohistochemistry, postmortem, autopsy

## Abstract

On 11 March 2020, the World Health Organization (WHO) declared a pandemic due to the spread of COVID-19 from Wuhan, China, causing high mortality rates all over the world. The related disease, which mainly affects the lungs, is responsible for the onset of Diffuse Alveolar Damage (DAD) and a hypercoagulability state, frequently leading to Severe Acute Respiratory Syndrome (SARS) and multiorgan failure, particularly in old and severe-critically ill patients. In order to find effective therapeutic strategies, many efforts have been made aiming to shed light on the pathophysiology of COVID-19 disease. Moreover, following the late advent of vaccination campaigns, the need for the comprehension of the pathophysiology of the fatal, although rare, thrombotic adverse events has become mandatory as well. The achievement of such purposes needs a multidisciplinary approach, depending on a correct interpretation of clinical, biochemical, biomolecular, and forensic findings. In this scenario, autopsies have helped in defining, on both gross and histologic examinations, the main changes to which the affected organs undergo and the role in assessing whether a patient is dead “from” or “with” COVID-19, not to mention whether the existence of a causal link exists between vaccination and thrombotic adverse events. In the present work, we explored the role of postmortem immunohistochemistry, and the increasingly used ancillary technique, in helping to understand the mechanism underlying the pathophysiology of both COVID-19 disease and COVID-19 vaccine-related adverse and rare effects.

## 1. Introduction

Since its first reported outbreak in Wuhan, China, in December 2019, COVID-19 (also known as SARS-CoV-2) has rapidly spread throughout the globe, thus leading the World Health Organization (WHO) to declare a pandemic on 11 March 2020 [1]. The resulting infection mainly affects the lungs, resulting in a broad spectrum of clinical manifestations that range from asymptomatic or mild, flu-like forms to Severe Acute Respiratory Syndrome (SARS) with multiorgan failure [2,3,4,5], especially in older patients who present comorbidities (e.g., hypertension, obesity, diabetes, chronic obstructive pulmonary disease, coronary artery disease, and chronic kidney disease) [2,3]. Therefore, the COVID-19 pandemic represents not only a leading cause of death worldwide, but also a burden for the healthcare system both in terms of intensive care unit overload and the management of economic resources [3,4,5,6,7].

The need for a correct clinical management and specific therapeutic strategies has thus led to the production of several research works aiming to shed light on the mechanisms underlying the pathophysiology of COVID-19. In such a context, the role of postmortem investigations is of utmost importance due to the possibility not only to directly analyze the affected organs, but also to achieve a correct diagnosis, thus assessing whether a patient is dead “from” or “with” COVID-19. Despite the publication of recommendations and safety strategies to be adopted in cases of confirmed and/or suspected COVID-19 deaths, several countries have chosen not to perform autopsies, except for selected cases [5,6]; as a result, the exact pathophysiological mechanisms of COVID-19 infection have been partly understood [4,5,6].

According to current scientific knowledge, SARS-CoV-2 infection starts when the viral Spike glycoprotein (S) binds to the Angiotensin-Converting Enzyme 2 (ACE2), which is highly expressed on epithelial cells of the respiratory tract and endothelial cells; the subsequent fusion of the viral envelope with the host cell membrane activates, at first, an innate immune response mediated by both the inflammasome and NF-κB pathway [2], thus leading to the release of a great number of pro-inflammatory cytokines (the so-called “cytokine storm”) responsible for the following activation of the adaptive immune response that contributes to the onset of a hyperinflammation state evolving into Acute Respiratory Distress Syndrome (ARDS), prominent hypercoagulability, and multiorgan failure [2,3,5,6].

Within a postmortal setting, lungs are considered the mostly affected organ with evidence, on gross and histologic examinations, of Diffuse Alveolar Damage (DAD) and microthrombi in the pulmonary vessels [5,6].

Another important aspect is related to vaccines that represent the most important countermeasure to fight the COVID-19 pandemic. Two vaccines are adenoviral vector-based vaccines (COVID-19 Vaccine Janssen and COVID-19 Vaccine AstraZeneca (Vaxzevria, ChAdOx1-S, respectively)) [8] and one of those, the AstraZeneca vaccine, has been associated with severe vascular reactions sourcing diffidence among the people. The mechanism by which the vaccine caused the vascular complications is still being defined, however, seemingly associated with an inadequate immune response [9].

In these difficult contexts, the postmortem studies are also useful to help the scientific community to better understand both the COVID-19 pathophysiology and the pathological events underlying the vaccine-related vascular reaction. In such a context, additional reliance on ancillary techniques, such as immunohistochemistry [10,11,12], allows the identification of specific cells and/or mediators recruited at the inflammation site, thus contributing to a better definition of the pathophysiological events guiding the immune response. Hence, the aim of the present work was to highlight the role of the postmortem investigations in the comprehension of the pathophysiological events underlying COVID-19 infection and vaccine-induced adverse vascular reactions, specifically focusing on the immunohistochemical approach.

## 2. Inflammatory Infiltrate

According to the research works produced so far, COVID-19 infection mostly affects the lungs, evolving into Severe Acute Respiratory Distress Syndrome (ARDS) in critical cases. The corresponding histological findings are represented by Diffuse Alveolar Damage (DAD) at different stages (mainly exudative and proliferative), characterized by hyaline membranes, intra-alveolar and/or septal and/or interstitial oedema, intra-alveolar fibrinous exudate, inflammatory cell infiltrates, type 2 pneumocyte hyperplasia/activation, and squamous metaplasia [2,3,4,5,6,13,14,15,16,17,18,19,20,21,22,23,24,25]. As evidenced in several works, a further help to better define the immune cell infiltrates in the lungs has been provided by the association of immunohistochemical analyses to the postmortem investigations (Table 1).

In their work, Lupariello et al. [4] relied on immunohistochemistry in order to characterize the lymphocytic immune infiltrate in the lungs of two patients with COVID-19, revealing the presence of a diffuse perivascular recruitment of T lymphocytes (CD3^+^) and focal infiltrations of CD8^+^ T lymphocytes and NK (TIA1^+^) in vessels and perivascular spaces, but not B lymphocytes infiltrates. As well as lymphocytes, macrophages also play a key role in COVID-19-induced response, as highlighted in the works of both Conde et al. [13] and Suess et al. [14], where the immunohistochemical reactivity for CD68 revealed the presence of intra-alveolar macrophage infiltrates, showing viral cytopathic-like changes, such as vesicular nuclei with prominent nucleoli, together with occasional multinucleated giant cells. The authors also performed immunohistochemistry for cytokeratin AE1/AE3 [13] and TTF-1 [13,14], which showed the same cytopathic changes, along with severe hyperplasia and type 2 pneumocytes. Similar results have been obtained by Barton et al. [15], who carried out immunohistochemical assays for both macrophage and lymphocytic markers on lung samples obtained from two patients positive to COVID-19. In both cases, the CD68 immunoreactivity revealed the presence of alveolar macrophages. The assessment of the immunoreactivity for CD3, CD4, and CD8 revealed the presence of T lymphocytes within the alveolar septa, with CD8^+^ T cells slightly outnumbering the CD4^+^ ones; in this case, CD20^+^ B lymphocytes, although rare, were also detected. In the second case, where acute bronchopneumonia *foci* along with aspirated food particles were detected, neutrophils and histiocytes were also found in the peribronchiolar airspaces.

In contrast with the works mentioned above, the evaluation by Cipolloni et al. [16] of the same markers in order to define the COVID-19-related immune infiltrates in the lungs of two cases revealed CD20^+^ B lymphocytes (specifically, CD79^+^ plasma cells) as the major lymphocytic infiltrate in both cases, together with CD68^+^ macrophages. CD4^+^ and CD8^+^ T cells were also detected, predominantly located in the interstitial spaces and around larger bronchioles.

The assessment of mostly the same immunohistochemical markers was carried out by Hanley et al. [17], who summarized the main findings from ten cases. Interstitial CD68^+^ macrophages were prominent in all cases; mild to moderate lymphocytic infiltrates were also detected in all ten cases, with CD4^+^ T cells outnumbering CD8^+^ T cells. Occasionally, CD56^+^ NK cells and small CD20^+^ B cells were found. Mild interstitial neutrophilic infiltrates were detected in 3/10 patients.

CD68^+^ macrophages and CD3^+^ T cells are considered as the main infiltrates within the alveolar spaces in the case discussed by Cîrstea et al. [18], along with very rare CD20^+^ B cells. The immunostaining for pan-cytokeratin (CK) AE1/AE3 or the CK7 revealed extensively proliferated, thickened, and detached epithelial cells; in addition, α-SMA^+^ myofibroblasts were mainly detected within the thickened alveolar spaces.

Interstitial lympho-monocytic infiltrates, with a predominance of CD3^+^ T lymphocytes over monocytes and the absence of CD20^+^ B lymphocytes, were the main immunohistochemical findings in the case analyzed in Aguiar et al.’s [19] work; the immunohistochemical investigations carried out by Oprinca et al. [20] in the three cases analyzed also showed focal areas of CD3^+^ and CD5^+^ T lymphocyte infiltrates, along with scattered CD20^+^ B lymphocytes; focal neutrophils were also detected. Immunohistochemistry was further performed for pancytokeratin panels (CKAE1–AE3; CK-MNF116), which were found positive within hyaline membranes, thus confirming their origin from the epithelial lining; finally, positive immunoreactivity for CK7 was found in pneumocytes that underwent viral cytopathic effects.

In the 10 cases evaluated in Fox et al.’s work [21], the inflammatory infiltrate was mainly represented by CD4^+^ and CD8^+^ T lymphocytes, predominantly detected within the interstitial spaces and around larger bronchioles and blood vessels. CD4^+^ T lymphocytes appeared in aggregates surrounding small vessels, in some of which platelets and small thrombi were also detected. In this case also, desquamated type 2 pneumocytes showing viral cytopathic effects (cytomegaly, enlarged nuclei) were present.

In the work by Duarte-Neto et al. [22], the immunohistochemical assay carried out on lung samples obtained from 10 COVID-19 cases revealed a difference in the abundance of both CD4^+^ and CD8^+^ T lymphocytes depending on the DAD phase: few CD20^+^ B lymphocytes were observed in all cases, while CD4^+^ and CD8^+^ T lymphocyte infiltrates ranged from scarce in cases with exudative DAD, to moderate in cases with fibroproliferative DAD. CD57^+^ NK cells were in all cases, while CD68^+^ macrophages were mostly distributed in the alveolar spaces and within fibroproliferative areas.

The evaluation of the immune infiltrate in the lungs of the 38 cases evaluated by Carsana et al. [23] highlighted the presence of a large number of CD68^+^ macrophages, mainly localized in the alveolar lumen, in 24 cases, while CD45^+^ and CD3^+^ T lymphocytes represented the main infiltrate within the interstitial space in 31 cases. The case series was further widened in the work from Bussani et al. [24], who carried out immunohistochemical evaluations of the inflammatory infiltrate in the lungs of 41 patients. The infiltrates mainly consisted of clusters of macrophages and CD8^+^ T lymphocytes outnumbering CD4^+^ cells.

Lastly, Ackermann et al. [25] compared the pulmonary immune infiltrates between a group of patients dead from COVID-19 and a group of patients dead from ARDS secondary to influenza A (H1N1) infection. They found a similar infiltrate of CD3^+^ T cells within precapillary and postcapillary vessel walls; among these, CD4^+^ T cells were revealed as more numerous in the lungs from patients with COVID-19 than in those from patients with influenza, whereas CD8^+^ T cells were less numerous. Sporadic CD15^+^ neutrophils were also detected adjacent to the alveolar epithelial lining in the COVID-19 group.

**Figure 1:**
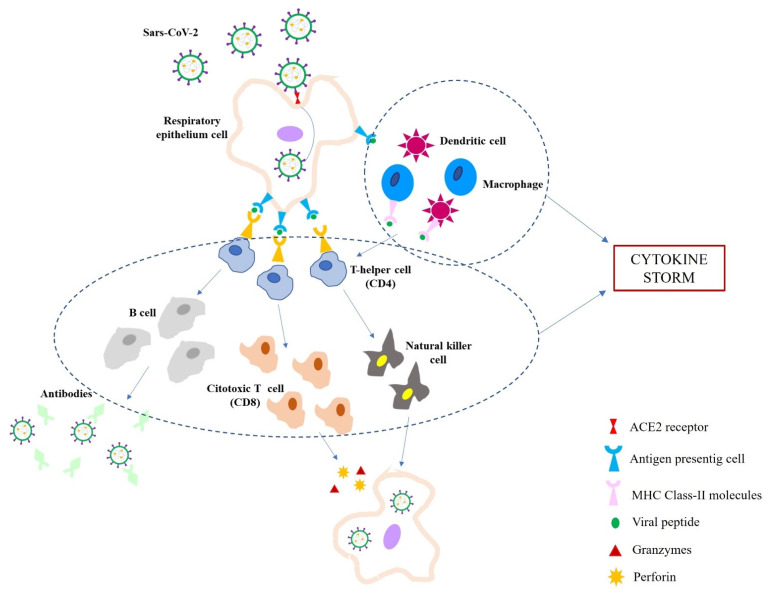
Schematic representation of Sars-CoV-2 infection immune response. Sars-CoV-2 enters the respiratory epithelium cell by the interaction of spike protein and ACE2 receptors; the virus is engulfed by APCs (antigen protein cells), which present viral antigens to T helper cells; dendritic cells and macrophages also recognize the whole virus and virus particle through MHC Class-II molecules and present viral peptides to T helper cells; T helper cells activate cytotoxic T cells and stimulate B cells; cytotoxic T cells produce perforin and granzymes to destroy the infected cell; stimulated B cells produce antibodies against Sars-CoV-2. Moreover, the activation of large numbers of white blood cells (T cells, B cells, NK cells, macrophages, dendritic cells, etc.) and resident tissue cells (i.e., epithelial and endothelial cells) determine the release of high numbers of pro-inflammatory cytokines (i.e., IL1B, IFNγ, IP10, and MCP-1) responsible for the so-called cytokine storm.

These findings are in accordance with the activation of a classic respiratory virus-like infection [2,5], which is mainly a cytotoxic response (Figure 1). The infection of the respiratory epithelial cells by SARS-CoV-2 induces a direct or indirect damage, as highlighted by the evidence of cytopathic changes such as hyperplastic, dysmorphic, and/or multinucleated type 2 pneumocytes [13,14,16,18,20,21,22,23]. As Antigen-Presenting Cells (APCs), the infected epithelial cells subsequently activate CD8^+^ T lymphocytes, which show their cytotoxic effect by releasing perforin and granzymes, thus inducing apoptosis; the cytotoxic response is also testified by the sporadic involvement of NK cells [4,17,22]. On the other hand, subepithelial dendritic cells (DCs) and alveolar macrophages recognize and present viral antigens to CD4^+^ T lymphocytes, subsequently inducing a Th_1_ and Th_17_ immune response polarization. The following interaction of CD4^+^ T cells with B lymphocytes promotes the production of IgM, IgA, and IgG isotype virus-specific antibodies [2,4,5].

In multiple studies, a reduction in peripheral blood of both CD4^+^ and CD8^+^ T cells has been observed, which mainly occurred in moderate and severe cases, also showing a correlation with SARS-CoV-2-related severity and mortality. Such a finding has then led to the postulation that the massive recruitment of T lymphocytes within the lungs (intra-alveolar lumen; alveolar septa; interstitium; perivascular) could explain the presence of lymphopenia in severe forms, thus contributing to the progression of SARS-CoV-2 infection [2,3,4,5,6,13,14,15,16,17,18,19,20,21,22,23,24].

As for the role of neutrophils, as they have been detected in cases where bacterial superinfection was also present [15,17,20,25,26], they do not seem to be directly involved in the COVID-19-related immune response.

Taken together, these results support the role of postmortem immunohistochemical investigations in helping define the COVID-19-related immune infiltrates [5].

## 3. Endothelial Injury, Thrombosis, and Complement Role

The evidence of platelet-rich thrombi and megakaryocytes in alveolar capillaries and small- and medium-sized pulmonary vessels [3,4,13,14,15,16,17,20,22,23,24,25], as well as thromboembolism of the main pulmonary arteries [18,27], has led to the hypothesis that both direct COVID-19 infection of the endothelial cells or the subsequent massive inflammatory response may contribute to the damage of the endothelium, thus inducing vasculopathy and endotheliitis, which further enhance the pulmonary damage [2,5,27,28,29,30]. In such a context, it has been postulated that a better understanding of the mechanisms underlying COVID-19-related vasculopathy could represent a rationale for therapies aimed to stabilize the endothelium, especially in vulnerable patients with pre-existing endothelial dysfunction (hypertension, diabetes, cardiovascular disease, etc.) [29].

The endothelial damage of pulmonary vessels in patients with COVID-19 has been immunohistochemically demonstrated on samples obtained postmortem only in a few works (Table 2).

In the three cases analyzed by Cîrstea et al. [18], the immunoreactivity to CD31+ and CD34+ endothelial cells and to collagen IV on the basal membranes revealed fragmented and discontinuous vascular profiles, in which context thrombi were also detected.

In Bussani et al.’s work [24], vasculitis of pulmonary macro- and microvasculature was histologically detected in 10/41 cases analyzed. Of the 41 cases, 11 were immunohistochemically assayed for the COVID-19 spike protein and the endothelial activation and disfunction markers CD142 (tissue factor), CD62 (E-Selectin), and VCAM-1 (adhesion molecule). In all cases, the samples showed immunoreactivity to the above-mentioned markers, along with evidence of endothelial alterations and macro- and microvascular thrombosis.

The evidence of massively high levels of a subset of cytokines—such as IL-6 and TNF-α—in critically ill patients has led to the hypothesis that, in the setting of the COVID-19-related cytokine storm, they could have a major role in enhancing the endothelial damage [28]. Such an hypothesis has been supported by a couple of works in which the supposedly involved cytokines were immunohistochemically assayed on lung samples.

Cipolloni et al. [16] carried out immunohistochemical investigations on samples obtained from two patients dead from COVID-19, and they compared the results to those found in a control patient (a 1-month-old newborn). The findings showed that in both cases—but not in the control case—endothelial cells were infected by COVID-19 (positive immunoreactivity to COVID nucleocapsid), while thrombosis activation was demonstrated by the positive immunoreactivity to factor VIII; within the affected vessels, a positive signal to TNF-α and IL-6 was also detected.

A similar investigation carried out by Nagashima et al. [31] on postmortem lung biopsies of six COVID-19 cases showed remarkably high levels on TNF-α in alveolar septal cells and alveolar capillary cells, and high levels of IL-6, ICAM-1, and caspase-1 in the endothelial cells. Based on these results, the authors postulated that the cytokine storm that follows SARS-CoV-2 infection induces a breakdown of the endothelial glycocalyx—which normally acts as a barrier against platelet activation—thus inducing endothelial dysfunction, endotheliitis, and thrombotic events. They further postulated that the activation of caspase-1 may contribute—via activation of the inflammasome—to a further release of other pro-inflammatory cytokines, such as IL-1β and IL-18, within capillary-alveolar endothelial cells.

In their work, Magro et al. [32] also found increased levels of several cytokines related to severe COVID-19 forms (IL-6, TNF-α, IL-1β, IL-8, p38, and INF-γ) in the lungs within areas of viral proliferation; an increase in caspase-3 and programmed death-ligand 1 (PDL1) was also observed in the pulmonary endothelia containing infectious virus.

Direct evidence of platelet-rich thrombi was highlighted in a few works in which CD61 immunostaining has been performed, such as in Lupariello et al.’s work [4], in which a diffuse platelet aggregation was highlighted in small- and medium-sized pulmonary vessels. In Hanley et al.’s work [17], CD61 immunostaining revealed macro- and microscopic thromboemboli, along with platelet- and fibrin-rich thrombi in alveolar capillaries, and small and medium pulmonary vessels. Fox et al. [21] reported evidence of both platelet-producing megakaryocytes and microthrombi within the alveolar capillaries. Along with platelet-fibrin thrombi, Carsana et al. [23] observed an increase in alveolar capillary megakaryocytes in 33/38 cases analyzed. Similar results were obtained by Rapkiewicz et al. [33] and Menter et al. [34]. The first ones found thrombi in pulmonary large and small vessels, while platelets were detected within the alveolar capillaries; in addition, an increase in megakaryocytes within the pulmonary microvasculature was also observed. As for Menter et al.’s work [34], in 5/11 cases in which immunohistochemistry for fibrin was performed, microthrombi were detected in alveolar capillaries.

A possible correlation between endothelial damage and complement activation via the alternative pathway (AP) has also been postulated [2,5,6,28]. Such a linkage has been assessed in an interesting work from Magro et al. [35] in which the authors detected a positive immunoreactivity to C5b-9 (membrane attack complex, MAC), C3d, and C4d in the inter-alveolar septal capillaries of all five cases analyzed, where they co-localized with anti-COVID spike protein antibodies. Furthermore, based on the discovery that mannose binding lectin (MBL) binds to the SARS-CoV-2 spike glycoprotein, the authors also postulated a possible involvement of the lectin pathway (LP) which, with a positive feedback loop, contributes to sustaining the alternative pathway activation, further enhancing endothelial damage and activation of the coagulation cascade.

## 4. Extra-Pulmonary Involvement

Both in clinical and postmortem settings, severe forms of COVID-19 infection frequently show a multi-organ involvement, although most findings are almost unspecific, thus making it challenging to understand whether their involvement depends on a direct damage, on an excessively impaired immune response, or on thrombosis-related ischemia [2,3,5]. The detection of low SARS-CoV-2 levels in organs such as the heart, liver, kidneys, and brain is consistent with a secondary involvement due to the ubiquitous expression of the ACE2 receptor [3]. Duarte-Neto et al. [22] classified the extra-pulmonary findings in three groups according to the possible cause: 1. findings due to comorbidities (myocardial hypertrophy and fibrosis; coronary artery disease; renal atherosclerosis; liver steatosis) [15,17,20,24,26,34]; 2. shock-related findings (acute tubular necrosis; centrilobular congestion) [18,20,24,26,29,34]; 3. findings of uncertain etiology (i.e., secondary to infection by SARS-CoV-2, systemic inflammation, or shock, such as leukocytic infiltrates and thrombosis) [4,17,18,20,21,22,24,26,32,33,34].

Magro et al. [32] carried out an immunohistochemical assessment for the deposition of C5b-9, C3d, and C4d on the lung (data previously reported), heart, liver, kidney, brain, and skin from 12 cases. The results revealed a significant endothelial and subendothelial microvascular deposition of C3d, C4d, and/or C5b-9 in all cases. Endothelial damage-related cytokines (IL6, TNF-α, IL-1β, IL-8, and p38) were also assessed, showing a significant increase in the microvascular extra-pulmonary endothelia where they strongly co-localized with both the viral spike protein and ACE2 receptor, including the skin and brain.

Finally, Rapkiewicz et al. [33] highlighted CD61^+^ platelet-rich thrombi also in the hepatic, renal, and cardiac microvasculature in all cases tested. Megakaryocytes were additionally observed in the cardiac and glomerular microvasculature.

## 5. Vaccine-Induced Thrombotic Thrombocytopenia

In order to front the COVID-19 pandemic, between December 2020 and January 2021, four vaccines have been authorized by the European Community: BNT162b2 (Pfizer–BioNTech); mRNA-1273 (Moderna); ChAdOx1 nCov-19 (AstraZeneca); COVID-19 Vaccine Janssen (Johnson & Johnson). Despite a positive ratio between benefits and risks, the vaccination with ChAdOx1 nCov-19 has been a source of diffidence because of the development—within one to three weeks following vaccination—of severe vascular adverse reactions temporally related to the vaccine administration (according to the EMA report of 7 April 2021: 169 cases of cerebral veins thrombosis, 53 cases of abdominal veins thrombosis, and 18 fatal cases, on around 34 million vaccinated people in the EEA and UK [36,37]).

In order to produce a workflow aimed to define the relationship between Adverse Events Following Immunization (AEFI) and COVID-19 vaccination, Pomara et al. [36] carried out postmortem investigations on two otherwise healthy subjects, respectively, dead after 19 and 24 days following vaccination with ChAdOx1 nCov-19. Prior to death, on admittance to the Emergency Department, both presented severe thrombocytopenia, low plasma fibrinogen, and very high levels of D-dimer; on CT examination, case 1 showed occlusive portal vein thrombosis with smaller thrombi in the splenic and upper mesenteric veins, and a massive intracerebral hemorrhage, while case 2 showed a very large intracranial hemorrhage. Along with gross and histologic investigations—which confirmed the antemortem CT findings—the authors also performed immunohistochemistry in order to characterize the immune infiltrates (CD163, CD66b), evaluate the presence of the adhesion molecule VCAM-1, and verify the activation of the complement pathway (C1r, C4d) and the deposition of anti-Platelet Factor 4 (PF4) antibodies. Strong positivity for adhesion molecules (VCAM-1), activated inflammatory cells (CD66b^+^, CD163, CD61^+^) expressing the complement fraction C1r, and anti-PF4/polyanion-antibodies was detected on vascular and perivascular tissues of the heart, lung, liver, kidney, ileum, and deep veins. Such findings not only led the authors to confirm a causal link between vaccination with ChAdOx1 nCov-19 and the development of immune thrombocytopenia mediated by platelet-activating antibodies against Platelet Factor 4 (PF4), but also allowed the production of a few hypotheses on its pathogenicity. Among these, the most credited is that of the production of antibodies that recognize Platelet Factor 4—involved in blood clot formation—probably following the exposure to polyanionic substances that are part of the vaccine composition, thus mimicking a heparin-induced autoimmune thrombocytopenia (HIT).

The above considerations integrate the research in clinical settings performed on patients with severe vascular adverse reactions to the ChAdOx1 nCov-19 vaccine [38,39]. In fact, the more shared evaluation regarding the pathogenesis of vaccine-induced immune thrombotic thrombocytopenia (VITT) suggests that an adenoviral vector vaccine can trigger an immune response leading to highly reactive anti-PF4 (anti-platelet factor 4) antibodies activating platelets through their FcγRIIa receptors (CD32) [40,41,42,43]. Particularly, this receptor is distributed on several cells such as platelets, monocytes, macrophages, neutrophils, natural killer cells, and endothelial cells, and studies described specific CD32 polymorphism (i.e., 131 Arg-His heterozygous or 131-His-His homozygous phenotypes) associated with an enhanced activation of platelets in HIT [44].

**Figure 2:**
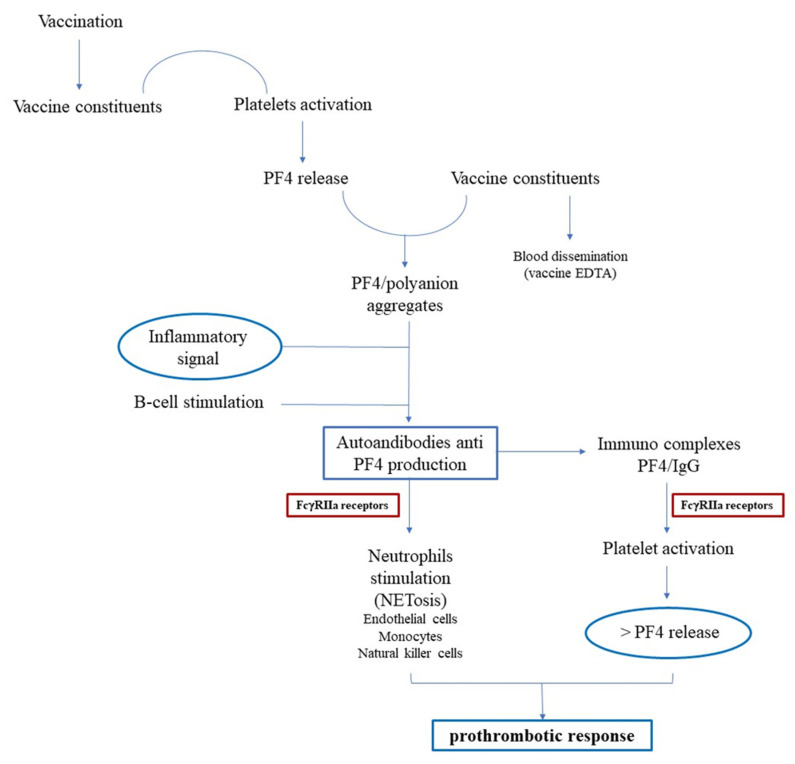
Schematic representation of suggested vaccine adverse vascular reaction. Vaccine constituents interact with platelets, determining platelet activation and PF4 release; PF4 binds vaccine constituents (possibly disseminated in blood for EDTA-related capillary leakage) forming aggregates; an inflammatory signal is generated with production of IgG anti-PF4-forming immuno-complexes PF4 (with vaccine constituents) and IgG; the immune complexes both stimulate neutrophils (and, possibly, endothelial cells, monocytes, and natural killer cells) and activate platelets releasing additional PF4, together determining a prothrombotic response.

Greinacher et al. [45,46] suggested the sequence of events (Figure 2) starting through the interaction of vaccine constituents (i.e., polyanions, such as glycosaminoglycans, polyphosphates, or DNA) with platelets resulting in platelet activation that release PF4; PF4 binds vaccine constituents forming multimolecular aggregates; vaccine EDTA increases capillary leakage and vascular permeability with blood dissemination of vaccine proteins (i.e., virus proteins, human originating proteins); an inflammatory signal is generated, stimulating the immune response, probably associated with immune complexes (vaccine constituents including its complexes with PF4 and preformed natural IgG); antibody anti-PF4 production is promoted by preformed B-cells stimulation; PF4/IgG immune complexes activate platelets releasing additional PF4, together with crosstalk with neutrophils determining NETosis and a prothrombotic response. Moreover, on the basis of previous studies describing the HIT pathogenicity, it can be supposed that, in addition to platelets, antibodies can induce activation of endothelial cells, natural killer cells, and monocytes releasing tissue factor (TF), which contribute to thrombosis [47,48].

## 6. Conclusions

Since its outbreak, COVID-19 has rapidly spread throughout the world, causing high mortality and morbidity rates [49]. The knowledge of the mechanisms used by the virus to infect the target cells has recently led to the production and distribution of several vaccines as a prevention strategy [36]. Nonetheless, also due to a partial adhesion to the vaccination campaign, COVID-19 has continued to affect thousands of people worldwide, also causing the emergence of stronger variants.

In light of this, an accurate comprehension of the pathophysiological mechanisms underlying the evolution of COVID-19 disease, especially in critical patients, is of utmost importance in order to find effective treatment strategies. To this end, a multidisciplinary approach is needed, comprehensive of clinical, biochemical, radiologic, biomolecular, and forensic investigations, each representing a piece of the whole puzzle [50,51,52].

Within a forensic context, autopsies have proved helpful in achieving a correct diagnosis in otherwise uncertain cases, assessing whether a patient was dead “from” or “with” COVID-19, thus providing reliable epidemiological, pathological, and global health data [5]. Furthermore, the possibility to directly analyze each single organ has helped identify the main pathological changes induced by the viral infection, namely DAD and thrombotic macro- and microangiopathy.

Based on the summarized results, the reliance on immunohistochemistry as an ancillary technique in a postmortal setting has proved useful in the comprehension of the main cellular infiltrates and mediators recruited in the mostly affected organs—the lungs. The comparison of these results with the findings from other works, carried out in a clinical setting or in which biochemical/biomolecular techniques were used, allowed the comprehension of the main pathophysiological mechanism of COVID-19 infection, namely the onset of a hyperinflammation state mediated by a cytokine storm-induced cytotoxic and T helper response primarily affecting the pulmonary parenchyma and vasculature; the massive activation of the immune system and microvascular damage might also be responsible for the indirect damage caused to other organs, even if a direct viral effect cannot be excluded. Such aspects, further investigated at a molecular level, could represent the target for selected therapies aimed to either block the virus entry into the target cells, shut the hyperimmune response, or stabilize the endothelium/inhibit platelet activation and aggregation.

Lastly, the immunohistochemical evidence of anti-PF4 antibodies’ co-localization with inflammatory cells, platelet, and complement mediators in patients dead for thrombotic complications following ChAdOx1 nCov-19 administration integrates the clinical evidence contributing to improve the knowledge on the pathophysiology of VITT. However, an increase in case studies is needed for a better definition of such mechanisms, also evaluating the possible relation between subject propensity to develop the VITT and genetic factors (i.e., polymorphism of CD32).

In conclusion, postmortem research, along with clinical studies, is a useful tool for understanding the pathogenesis and pathophysiology of both COVID-19 and VITT, making a contribution in order to benefit global public health.

## Figures and Tables

**Table 1:** Summary of immunohistochemical markers for lung inflammatory infiltrate.

Authors	*n*. Sample	Markers
Lupariello et al. [4]	*n*. 2	positivity of CD3, CD8, NK (TIA1); negativity of B lymphocytes
Navarro Conde et al. [13]	*n*. 1	positivity of CD68, cytokeratin AE1/AE3, TTF-1
Suess et al. [14]	*n*. 1	positivity of CD68, cytokeratin AE1/AE3, TTF-1
Barton et al. [15]	*n*. 2	positivity of CD68, CD3, CD4, CD8, CD20 (rare)
Cipolloni et al. [16]	*n*. 2	positivity of CD68, CD20/CD79, CD4, CD8
Hanley et al. [17]	*n*. 10	positivity of CD68, CD4, CD8, CD56, CD20
Cîrstea et al. [18]	*n*. 1	positivity of CD68, CD3, CD20, cytokeratin AE1/AE3/7, α-SMA
Aguiar et al. [19]	*n*. 1	positivity of CD3 and CD20
Oprinca et al. [20]	*n*. 3	positivity of CD3, CD5, CD20, cytokeratin AE1/AE3/MNF116/7
Fox et al. [21]	*n*. 10	positivity of CD4, CD8
Nunes Duarte-Nerto et al. [22]	*n*. 10	positivity of CD4, CD8, CD20, CD68, CD57
Carsana et al. [23]	*n*. 38	positivity of CD3, CD45, CD68
Bussani et al. [24]	*n*. 41	positivity of CD4, CD8
Ackermann et al. [25]	*n*. 7	positivity of CD3, CD4, CD8, CD15

**Table 2:** Summary of immunohistochemical marker for lung endothelial damage and thromboembolism.

Authors	*n*. Sample	Markers
Cîrstea et al. [18]	*n*. 1	positivity of CD31, CD34, collagen IV (fragmentation of vascular profiles)
Bussani et al. [24]	*n*. 11	positivity of CD42, CD62, VCAM-1
Cipolloni et al. [16]	*n*. 2	positivity of factor VIII, TNF-α, IL-6
Nagashima et al. [31]	*n*. 6	positivity of TNF-α, IL-6, ICAM-1, caspase-1
Magro et al. [32]	*n*. 12	positivity of TNF-α, IL-6, IL-1β, IL-8, p38, INF-γ, caspase-3, PDL1
Lupariello et al. [4]	*n*. 2	positivity of CD61
Hanley et al. [17]	*n*. 10	positivity of CD61
Fox et al. [21]	*n*. 10	positivity of CD4, CD8, CD31
Carsana et al. [23]	*n*. 33	positivity of CD61
Rapkiewicz et al. [33]	*n*. 7	positivity of CD61
Menter et.al. [34]	*n*. 4	positivity of fibrin
Magro et al. [35]	*n*. 5	positivity of C5b-9, C3d, C4d

## Data Availability

Data supporting reported results can be found in the work.
